# Performance of an Antigen-Based Test for Asymptomatic and Symptomatic SARS-CoV-2 Testing at Two University Campuses — Wisconsin, September–October 2020

**DOI:** 10.15585/mmwr.mm695152a3

**Published:** 2021-01-01

**Authors:** Ian W. Pray, Laura Ford, Devlin Cole, Christine Lee, John Paul Bigouette, Glen R. Abedi, Dena Bushman, Miranda J. Delahoy, Dustin Currie, Blake Cherney, Marie Kirby, Geroncio Fajardo, Motria Caudill, Kimberly Langolf, Juliana Kahrs, Patrick Kelly, Collin Pitts, Ailam Lim, Nicole Aulik, Azaibi Tamin, Jennifer L. Harcourt, Krista Queen, Jing Zhang, Brett Whitaker, Hannah Browne, Magdalena Medrzycki, Patricia Shewmaker, Jennifer Folster, Bettina Bankamp, Michael D. Bowen, Natalie J. Thornburg, Kimberly Goffard, Brandi Limbago, Allen Bateman, Jacqueline E. Tate, Douglas Gieryn, Hannah L. Kirking, Ryan Westergaard, Marie Killerby, CDC COVID-19 Surge Laboratory Group, CDC COVID-19 Surge Laboratory Group, Baoming Jiang, Jan Vinjé, Amy L. Hopkins, Eric Katz, Leslie Barclay, Mathew Esona, Rashi Gautam, Slavica Mijatovic-Rustempasic, Sung-Sil Moon, Theresa Bessey, Preeti Chhabra, Sarah L. Smart, Raydel Anderson, Kay W. Radford, Gimin Kim, Dexter Thompson, Congrong Miao, Min-hsin Chen, Lalitha Gade, Renee Galloway, Kashif Sahibzada, Nhien M. Tran, Srinivasan Velusamy, HaoQiang Zheng, Kenny Nguyen, Claire Hartloge, Brent Jenkins, Phili Wong

**Affiliations:** ^1^CDC COVID-19 Response Team; ^2^Epidemic Intelligence Service, CDC; ^3^Wisconsin Department of Health Services; ^4^School of Medicine and Public Health, University of Wisconsin-Madison; ^5^Laboratory Leadership Service, CDC; ^6^Agency for Toxic Substances and Disease Registry, Atlanta, Georgia; ^7^University of Wisconsin-Oshkosh; ^8^University Health Services, University of Wisconsin-Madison; ^9^Wisconsin Veterinary Diagnostic Laboratory, University of Wisconsin-Madison; ^10^Winnebago County Health Department, Oshkosh, Wisconsin; ^11^Wisconsin State Laboratory of Hygiene.; CDC; CDC; CDC; CDC; CDC; CDC; CDC; CDC; CDC; CDC; CDC; CDC; CDC; CDC; CDC; CDC; CDC; CDC; CDC; CDC; CDC; CDC; CDC; CDC; Oak Ridge Institute for Science and Education, Oak Ridge, Tennessee; Oak Ridge Institute for Science and Education, Oak Ridge, Tennessee; Oak Ridge Institute for Science and Education, Oak Ridge, Tennessee; Oak Ridge Institute for Science and Education, Oak Ridge, Tennessee

Antigen-based tests for SARS-CoV-2, the virus that causes coronavirus disease 2019 (COVID-19), are inexpensive and can return results within 15 minutes (*1*). Antigen tests have received Food and Drug Administration (FDA) Emergency Use Authorization (EUA) for use in asymptomatic and symptomatic persons within the first 5–12 days after symptom onset (*2*). These tests have been used at U.S. colleges and universities and other congregate settings (e.g., nursing homes and correctional and detention facilities), where serial testing of asymptomatic persons might facilitate early case identification (*3*–*5*). However, test performance data from symptomatic and asymptomatic persons are limited. This investigation evaluated performance of the Sofia SARS Antigen Fluorescent Immunoassay (FIA) (Quidel Corporation) compared with real-time reverse transcription–polymerase chain reaction (RT-PCR) for SARS-CoV-2 detection among asymptomatic and symptomatic persons at two universities in Wisconsin. During September 28–October 9, a total of 1,098 paired nasal swabs were tested using the Sofia SARS Antigen FIA and real-time RT-PCR. Virus culture was attempted on all antigen-positive or real-time RT-PCR–positive specimens. Among 871 (79%) paired swabs from asymptomatic participants, the antigen test sensitivity was 41.2%, specificity was 98.4%, and in this population the estimated positive predictive value (PPV) was 33.3%, and negative predictive value (NPV) was 98.8%. Antigen test performance was improved among 227 (21%) paired swabs from participants who reported one or more symptoms at specimen collection (sensitivity = 80.0%; specificity = 98.9%; PPV = 94.1%; NPV = 95.9%). Virus was isolated from 34 (46.6%) of 73 antigen-positive or real-time RT-PCR–positive nasal swab specimens, including two of 18 that were antigen-negative and real-time RT-PCR–positive (false-negatives). The advantages of antigen tests such as low cost and rapid turnaround might allow for rapid identification of infectious persons. However, these advantages need to be balanced against lower sensitivity and lower PPV, especially among asymptomatic persons. Confirmatory testing with an FDA-authorized nucleic acid amplification test (NAAT), such as RT-PCR, should be considered after negative antigen test results in symptomatic persons, and after positive antigen test results in asymptomatic persons (*1*).

Paired nasal swabs were collected from students, faculty, staff members, and other affiliates^†^ at two Wisconsin university campuses during university-based testing programs. At university A, all persons tested (screening or diagnostic) at the university testing center during October 1–9 were eligible to participate. At university B, only students who were quarantined during September 28–October 6 after exposure to persons with COVID-19 could participate.

All participants completed a questionnaire and provided information on demographic characteristics, current and past (14 days) symptoms,^§^ and recent exposure^¶^ to persons with COVID-19. For each participant, two mid-turbinate nasal swabs were collected by health care personnel at university A and were self-collected under supervision at university B. Both nostrils were sampled with each of the two swabs. Swabs for antigen testing were analyzed according to the manufacturer’s instructions.** Swabs for real-time RT-PCR were stored in viral transport media at 39°F (4°C) and analyzed within 24–72 hours of collection. At university A, real-time RT-PCR was performed using the CDC 2019-nCoV real-time RT-PCR diagnostic panel (*6*), with cycle threshold (Ct) values reported for the N1 and N2 viral nucleocapsid protein gene regions. At university B, real-time RT-PCR was performed using the TaqPath COVID-19 Combo Kit (Thermo Fisher Scientific). Viral culture^††^ (*7*) was attempted on residual RT-PCR specimens if the RT-PCR or antigen test result was positive.

Statistical analyses were performed using Stata (version 16.1; StataCorp). Sensitivity, specificity, PPV, and NPV were calculated for antigen testing compared with real-time RT-PCR results. Ninety-five percent confidence intervals (CIs) were calculated using the exact binomial method; t-tests were used for Ct value comparisons^§§^; p-values <0.05 were considered statistically significant. This investigation was reviewed by CDC and was conducted consistent with applicable federal law and CDC policy.^¶¶^ Ethical review boards at both universities determined the activity to be nonresearch public health surveillance (*2*).

Among a total of 1,105 total nasal swab pairs submitted, seven (0.06%) were excluded for having inconclusive antigen or real-time RT-PCR results. Test comparisons were performed on 1,098 paired nasal swabs (2,196 total swabs), including 1,051 pairs (95.7%) from university A and 47 pairs (4.3%) from university B (Table 1). Among the 1,098 pairs evaluated, 994 (90.5%) were provided by students aged 17–53 years (median = 19 years), 82 (7.5%) by university faculty or staff members aged 22–63 years (median = 38 years), and 22 (2.0%) by other university affiliates aged 15–64 years (median = 29 years). Fifty-seven persons participated more than once on different testing days. Overall, 453 (41.3%) participants were male, and 917 (83.5%) were non-Hispanic White. At specimen collection, 227 (20.7%) participants reported experiencing one or more COVID-19 symptoms, and 871 (79.3%) reported no symptoms.

**TABLE 1:** Characteristics and symptoms of persons providing paired nasal swabs (N = 1,098),* by results for SARS-CoV-2 real-time reverse transcription–polymerase chain reaction (RT-PCR) and Sofia SARS Antigen Fluorescent Immunoassay testing† — two universities, Wisconsin, September–October 2020

Characteristic	No (%)				
	True positives (N = 39)	False negatives (N = 18)	False positives (N = 16)	True negatives (N = 1,025)	Total (N = 1,098)
**Testing site**					
University A^§^	37 (94.9)	17 (94.4)	15 (93.8)	982 (95.8)	**1,051 (95.7)**
University B^¶^	2 (5.1)	1 (5.6)	1 (6.3)	43 (4.2)	**47 (4.3)**
**Sex**					
Male	16 (41.0)	9 (50.0)	12 (75.0)	416 (40.6)	**453 (41.3)**
Female	23 (59.0)	9 (50.0)	4 (25.0)	609 (59.4)	**645 (58.7)**
**Age group (yrs)**					
15–24**	35 (89.7)	16 (88.9)	11 (68.8)	909 (88.7)	**971 (88.4)**
≥25	4 (10.3)	2 (11.1)	5 (31.3)	116 (11.3)	**127 (11.6)**
**Race/Ethnicity^††^**					
White	31 (79.5)	17 (94.4)	12 (75.0)	857 (83.6)	**917 (83.5)**
Hispanic/Latino	6 (15.4)	0 (0)	1 (6.3)	54 (5.3)	**61 (5.6)**
Black/African-American	0 (0)	1 (5.6)	2 (12.5)	26 (2.5)	**29 (2.6)**
Asian/Pacific Islander	0 (0)	0 (0)	0 (0)	49 (4.8)	**49 (4.5)**
American Indian/Alaska Native	0 (0)	0 (0)	0 (0)	3 (0.3)	**3 (0.3)**
Other/Unknown/Multiple races	2 (5.1)	0 (0)	1 (6.3)	36 (3.5)	**39 (3.6)**
**University status**					
Student	35 (89.7)	17 (94.4)	13 (81.3)	929 (90.6)	**994 (90.5)**
Faculty or staff member	4 (10.3)	1 (5.6)	3 (18.8)	74 (7.2)	**82 (7.5)**
Other affiliate or unknown^§§^	0 (0)	0 (0)	0 (0)	22 (2.2)	**22 (2.0)**
**Exposure**^¶¶^ **to a COVID-19 case**					
Been in close contact in the past 14 days	13 (33.3)	9 (50.0)	4 (25.0)	128 (12.5)	**154 (14.0)**
**Quarantine status**					
Quarantined at time of specimen collection	17 (43.6)	6 (33.3)	3 (18.8)	109 (10.6)	**135 (12.3)**
Time between quarantine initiation to specimen collection, median days (range)	1 (0–8)	3.5 (0–6)	1 (0–4)	4 (0–28)	**4 (0–28)**
**Reported symptoms**					
**No current symptoms**	7 (17.9)	10 (55.6)	14 (87.5)	840 (82.0)	**871 (79.3)**
One or more symptoms in the past 14 days	2 (28.6)	1 (10.0)	0 (0)	50 (6.0)	**53 (6.1)**
No symptoms in the past 14 days	5 (71.4)	9 (90.0)	14 (100.0)	790 (94.0)	**818 (93.9)**
**One or more current symptoms**	32 (82.1)	8 (44.4)	2 (12.5)	185 (18.0)	227 (20.7)
Nasal congestion	24 (75.0)	2 (25.0)	1 (50.0)	87 (47.0)	**114 (50.2)**
Sore throat	12 (37.5)	5 (62.5)	1 (50.0)	79 (42.7)	**97 (42.7)**
Headache	17 (53.1)	3 (37.5)	1 (50.0)	66 (35.7)	**87 (38.3)**
Cough	18 (56.3)	6 (75.0)	1 (50.0)	45 (24.3)	**70 (30.8)**
Fatigue	14 (43.8)	3 (37.5)	1 (50.0)	42 (22.7)	**60 (26.4)**
Muscle aches	11 (34.4)	2 (25.0)	0 (0)	30 (16.2)	**43 (18.9)**
Shortness of breath	7 (21.9)	1 (12.5)	0 (0)	16 (8.6)	**24 (10.6)**
Chills	4 (12.5)	0 (0)	0 (0)	14 (7.6)	**18 (7.9)**
Diarrhea	3 (9.4)	0 (0)	0 (0)	15 (8.1)	**18 (7.9)**
Nausea or vomiting	3 (9.4)	0 (0)	0 (0)	14 (7.6)	**17 (7.5)**
Loss of taste	8 (25.0)	2 (25.0)	1 (50.0)	3 (1.6)	**14 (6.2)**
Loss of smell	8 (25.0)	2 (25.0)	1 (50.0)	2 (1.1)	**13 (5.7)**
Fever	6 (18.8)	0 (0)	0 (0)	5 (2.7)	**11 (4.8)**
Difficulty breathing	3 (9.4)	0 (0)	0 (0)	8 (4.3)	**11 (4.8)**
Abdominal pain	1 (3.1)	0 (0)	0 (0)	6 (3.2)	**7 (3.1)**
Rigors	0 (0)	0 (0)	0 (0)	0 (0)	**0 (0.0)**
Other reported symptoms***	1 (3.1)	0 (0)	0 (0)	4 (2.2)	**5 (2.2)**
**Symptom onset date reported**	31 (96.9)	8 (100.0)	2 (100.0)	169 (91.4)	**210 (92.5)**
≤5 days between reported symptom onset and specimen collection	23 (74.2)	8 (100.0)	1 (50.0)	120 (71.0)	**152 (72.4)**

* Includes 57 participants who received multiple tests and were included more than once in the analysis.

^†^ True positive = antigen-positive and real-time RT-PCR–positive; false negative = antigen-negative and real-time RT-PCR–positive; false positive = antigen-positive and real-time RT-PCR–negative; true negative = antigen-negative and real-time RT-PCR–negative; these definitions do not reflect results from viral culture.

^§^ At university A, real-time RT-PCR was performed using the CDC 2019-nCoV real-time RT-PCR diagnostic panel for detection of SARS-CoV-2.

^¶^ At university B, real-time RT-PCR was performed using Thermo Fisher Scientific’s TaqPath COVID-19 Combo Kit for detection of SARS-CoV-2.

** One university staff member’s child aged 15 years. All other participants were aged ≥17 years.

^††^ Non-Hispanic ethnicity represented for all White, Black/African-American, Asian/Pacific Islander, American Indian/Alaska Native, Other/Unknown/Multiple races.

^§§^ Other affiliates were participants who did not mark “student” or “staff” on the questionnaire (they selected “other” or did not respond); the majority of these persons were family members of staff members.

^¶¶^ Ever in close contact was defined as within 6 feet for ≥15 minutes of a person with a diagnosis of COVID-19.

*** Other reported symptoms included allergies, cough that is not dry, and difficulty breathing from anxiety.

Among 227 paired specimens from symptomatic participants, 34 (15.0%) were antigen-positive, and 40 (17.6%) were real-time RT-PCR-positive. The median interval from symptom onset to specimen collection was 3 days (interquartile range = 1–6 days; 7.5% missing). Among symptomatic participants, antigen testing sensitivity was 80.0% (32 of 40), specificity was 98.9% (185 of 187), PPV was 94.1% (32 of 34), and NPV was 95.9% (185 of 193) (Table 2). For specimens collected within 5 days of reported symptom onset (72.4%; 152 of 210), sensitivity was 74.2% (23 of 31), and specificity was 99.2% (120 of 121).

**TABLE 2:** Sensitivity, specificity, positive predictive value, and negative predictive value of Sofia SARS Antigen Fluorescent Immunoassay compared with real-time reverse transcription–polymerase chain reaction (RT-PCR) among asymptomatic and symptomatic persons — two universities, Wisconsin, September–October 2020

Antigen test result	Real-time RT-PCR result, no.					
	Asymptomatic (N = 871)			Symptomatic* (N = 227)		
	Positive	Negative	Total	Positive	Negative	Total
Positive	7	14	**21**	32	2	**34**
Negative	10	840	**850**	8	185	**193**
**Total**	**17**	**854**	**871**	**40**	**187**	**227**
**Test evaluation, % (95% CI)**						
Sensitivity	41.2 (18.4–67.1)			80.0 (64.4–90.9)		
Specificity	98.4 (97.3–99.1)			98.9 (96.2–99.9)		
Positive predictive value	33.3 (14.6–57.0)			94.1 (80.3–99.3)		
Negative predictive value	98.8 (97.8–99.4)			95.9 (92.0–98.2)		

**Abbreviation:** CI = confidence interval.

* One or more symptoms reported.

Among 871 paired specimens from asymptomatic participants, 21 (2.4%) were antigen-positive and 17 (2.0%) were real-time RT-PCR-positive. Antigen testing sensitivity was 41.2% (seven of 17), specificity was 98.4% (840 of 854), PPV was 33.3% (seven of 21), and NPV was 98.8% (840 of 850). Test performance was not significantly (p>0.05) different when excluding 53 (6.1%) of 871 participants who were asymptomatic at the time of testing but had reported one or more symptoms in the preceding 14 days.

Sixteen paired swabs were antigen-positive and real-time RT-PCR–negative (i.e., false-positive), including 14 (66.7%) of 21 positive antigen results from asymptomatic participants and two (5.9%) of 34 from symptomatic participants. Eight of the 16 false-positive results were recorded during a 1-hour period at university A. In this instance, a series of consecutive positive results in asymptomatic persons was noted, and investigators offered repeat antigen testing to the affected participants. Six of eight participants were reswabbed within 1 hour, and all six received negative test results on a second antigen test. All eight initial paired swabs from these participants were negative on real-time RT-PCR. Because no user errors could be identified, the false-positive results were included in analysis. Eighteen false-negative antigen test results were obtained, including 10 (58.8%) of 17 real-time RT-PCR–positive tests from asymptomatic participants, and eight (20.0%) of 40 from symptomatic participants. All false-negative results from symptomatic participants were from specimens collected <5 days after onset of symptoms (median = 2 days). Ct values for specimens with false-negative antigen results were significantly higher compared with antigen- and real-time RT-PCR-positive specimens (mean N1 Ct = 32.3 versus 23.7; p<0.01) (Figure).

**FIGURE:**
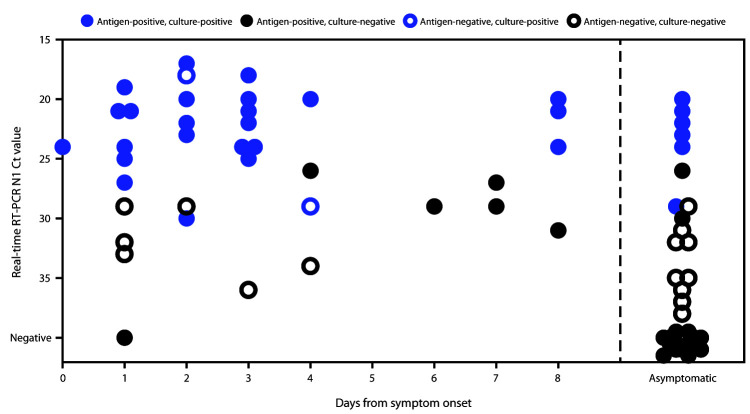
Viral culture results among participants with positive Sofia SARS Antigen Fluorescent Immunoassay or positive SARS-CoV-2 real-time reverse transcription–polymerase chain reaction (RT-PCR) results (n = 69),* by cycle threshold (Ct) value† and the interval between specimen collection and reported symptom onset or asymptomatic status — university A, Wisconsin, September–October 2020 * n = 30 antigen- and culture-positive; n = 22 antigen-positive and culture-negative; n = 15 antigen- and culture-negative; n = two antigen- negative and culture-positive. ^†^ Ct values represent cycle thresholds for the N1 target probe during SARS-CoV-2 real-time RT-PCR; Ct values are represented on the y-axis in descending order to indicate that lower Ct values represent higher levels of RNA in the specimen.

Virus was recovered from 34 (46.6%) of 73 positive specimens, including 32 (82.1%) of 39 specimens with concordant positive results and two (11.1%) of 18 with false-negative antigen results; no virus was recovered from 16 specimens with false-positive antigen test results. The two specimens with false-negative antigen results that were culture-positive were from two symptomatic participants who had specimens collected at day 2 and day 4 after symptom onset.***

## Discussion

The Sofia SARS Antigen FIA received FDA EUA on May 8, 2020, for use in symptomatic persons within 5 days of symptom onset (*2*). In this investigation, among persons reporting COVID-19–compatible symptoms at specimen collection, the test was less accurate (sensitivity = 80.0%; specificity = 98.9%) than reported in the FDA EUA (sensitivity = 96.7%; specificity = 100%) (*2*). Two of eight specimens from symptomatic persons that had false-negative antigen test results were positive by viral culture, indicating that potentially infectious persons might not be detected by antigen testing. To reduce the impact of false-negative antigen test results, confirmatory testing with an FDA-authorized NAAT, such as RT-PCR, should be considered following negative antigen test results in symptomatic persons (*1*).

Among asymptomatic participants, antigen test sensitivity was 41.2%, specificity was 98.4%, and PPV in this population was 33.3%. This low PPV was observed despite a relatively high prevalence of SARS-CoV-2 in this population (5.2% prevalence overall; 2.0% among asymptomatic persons), suggesting that PPV could be even lower when using this antigen test among populations with lower expected SARS-CoV-2 prevalence. To account for false-positive results when using antigen tests for asymptomatic screening, confirmatory NAAT testing should be considered following positive antigen test results in asymptomatic persons, particularly when pretest probability of SARS-CoV-2 infection is low (*1*). The NPV of antigen testing among asymptomatic participants was 98.8%, and virus was not cultured from asymptomatic participants with antigen-negative results, indicating that asymptomatic persons with negative antigen results are unlikely to be infected with SARS-CoV-2 and would not require confirmatory NAAT (*1*).

The findings in this report are subject to at least four limitations. First, participants were predominantly young adults in university settings where ongoing serial testing was being conducted. Antigen test performance might differ in other populations with different characteristics and testing schedules. Second, given the limitations of RT-PCR, some false-positive antigen test results might represent true infections not identified by RT-PCR. Third, the ability to recover infectious virus in culture is limited and decreases for specimens with higher Ct values (*8*); a lack of virus recovery by culture does not indicate that a person is not infectious. Finally, this investigation evaluated the Sofia SARS Antigen FIA, and cannot be generalized to other FDA-authorized SARS-CoV-2 antigen tests.

Serial testing of asymptomatic and symptomatic persons has been proposed for prevention and control of SARS-CoV-2 transmission (*9*,*10*) and is currently being implemented at U.S. colleges and universities and in other congregate settings (*3*–*5*). Despite reduced sensitivity compared with real-time RT-PCR, the use of antigen tests for serial testing in these settings, particularly when RT-PCR tests are not available or have a prolonged turnaround time, might still allow rapid identification of infectious persons and control of outbreaks (*1*). However, antigen-based testing strategies should account for the lower sensitivity and lower PPV when used for asymptomatic screening by considering confirmatory testing with an FDA-authorized NAAT, such as RT-PCR, after a positive antigen test result in an asymptomatic person. Confirmatory testing should also be considered following a negative antigen test result in a person experiencing COVID-19–compatible symptoms. All persons with negative antigen test results should continue to take measures to prevent SARS-CoV-2 transmission, including wearing a mask, reducing contact with nonhousehold members, and getting tested if they experience symptoms or have close contact with someone who has COVID-19.^†††^Symptomatic persons with negative antigen test results should continue to follow CDC guidance^§§§^ for persons who might have COVID-19, including staying home except to get medical care and protecting household members by staying in a separate room, wearing a mask indoors, washing hands often, and frequently disinfecting surfaces.

**Summary**

**What is already known about this topic?**

Antigen tests for SARS-CoV-2 are inexpensive and can return results within 15 minutes, but test performance data in asymptomatic and symptomatic persons are limited.

**What is added by this report?**

Compared with real-time reverse transcription–polymerase chain reaction (RT-PCR) testing, the Sofia antigen test had a sensitivity of 80.0% and specificity of 98.9% among symptomatic persons; accuracy was lower (sensitivity 41.2% and specificity 98.4%) when used for screening of asymptomatic persons.

**What are the implications for public health practice?**

To account for reduced antigen test accuracy, confirmatory testing with a nucleic acid amplification test (e.g., RT-PCR) should be considered after negative antigen test results in symptomatic persons and positive antigen test results in asymptomatic persons.

## Footnotes

* These authors contributed equally to this report.

† Other affiliates were participants who did not mark “student” or “staff” on the questionnaire (they selected “other” or did not respond); the majority of these persons were family members of staff members.

§ Symptom list was based on the interim position statement for COVID-19 case definitions from the Council of State and Territorial Epidemiologists, updated August 7, 2020. Clinical criteria for COVID-19 included fever, cough, shortness of breath, fatigue, sore throat, headache, muscle aches, chills, nasal congestion, difficulty breathing, diarrhea, nausea, vomiting, abdominal pain, rigors, loss of taste, and loss of smell. https://cdn.ymaws.com/www.cste.org/resource/resmgr/ps/positionstatement2020/Interim-20-ID-02_COVID-19.pdf.

¶ Recent exposure was defined as being within 6 feet of a person with a COVID-19 diagnosis for ≥15 minutes in the past 14 days.

** https://www.fda.gov/media/137885/download.

†† Specimens were used to perform a limiting-dilution inoculation of Vero CCL-81 cells, and cultures showing evidence of cytopathic effect (CPE) were tested by real-time RT-PCR for the presence of SARS-CoV-2 RNA. Viral recovery was defined as any culture in which the first passage had an N1 Ct at least twofold lower than the corresponding clinical specimen.

§§ Ct values from real-time RT-PCR were only compared for specimens collected at university A that were analyzed with the CDC 2019-nCoV real-time RT-PCR diagnostic panel for detection of SARS-CoV-2.

¶¶ 45 C.F.R. part 46.102(l)(2), 21 C.F.R. part 56; 42 U.S.C. Sect. 241(d); 5 U.S.C. Sect. 552a; 44 U.S.C. Sect. 3501 et seq.

*** The participant with a false-negative result 2 days after symptom onset had a repeat specimen 2 days later; the results of testing were positive by antigen test and by real-time RT-PCR.

††† https://www.cdc.gov/coronavirus/2019-ncov/prevent-getting-sick/prevention.html.

§§§ https://www.cdc.gov/coronavirus/2019-ncov/if-you-are-sick/steps-when-sick.html.
